# Physicians and Pharmacists

**Published:** 1882-02

**Authors:** 


### Editorial.

PHYSICIANS AND PHARMACISTS.— The short article in this number of THE JOURNAL AND EXAMINER, in which the practice of some physicians in signing certificates testifying to the qualities of medicinal agents and compounds, and of pharmacists in putting *trade marks* on their productions, is commended, has been admitted on the principle that both sides should be heard, and not because we agree with its sentiments. On the contrary, we think that both practices are in bad taste, and essentially vicious in their tendencies. N. S. D.

